# Coexpression and synexpression patterns across languages: comparative concepts and possible explanations

**DOI:** 10.3389/fpsyg.2023.1236853

**Published:** 2023-10-24

**Authors:** Martin Haspelmath

**Affiliations:** Max Planck Institute for Evolutionary Anthropology, Leipzig, Germany

**Keywords:** lexical typology, colexification, syncretism, cumulative exponence, polysemy

## Abstract

Meanings and linguistic shapes (or forms) do not always map onto each other in a unique way, and linguists have used all kinds of different terms for such situations: Ambiguity, polysemy, syncretism, lexicalization, semantic maps; portmanteau, cumulative exponence, feature bundling, underspecification, and so on. In the domain of lexical comparison, the term *colexification* has become generally established in recent years, and in the present paper, I extend this word-formation pattern in a regular way (*cogrammification*, *coexpression*; *syllexification*, *syngrammification*, *synexpression*). These novel terms allow us to chart the range of relevant phenomena in a systematic way across the grammar-lexicon continuum, and to ask whether highly general explanations of coexpression and synexpression patterns are possible. While there is no new proposal for explaining coexpression here, I will suggest that frequency of occurrence plays a crucial role in explaining synexpression patterns.

## 1. Introduction: coexpression and synexpression

This paper discusses and exemplifies a range of meaning–shape correspondence patterns in human languages, most of which have been extensively dealt with in the literature since the 1950s. What is (relatively) new here is (i) that they are treated together comprehensively in a uniform and transparent conceptual and terminological framework, and (ii) that I ask how the universal trends in such correspondence patterns might be explained in a general way.

The most important terms and concepts discussed in this paper are summarized in Table 1. *Coexpression* refers to the availability of two meanings for a minimal form in different contexts, while *synexpression* refers to the simultaneous presence of two meanings in a minimal form.

**Table 1:** The key concepts of this paper.

Coexpression	Examples
Colexification	German *Tasche* colexifies ‘bag’ and ‘pocket’
Cogrammification	Latin *-ae* cogrammifies ‘genitive’ and ‘dative’
Dislexification	English dislexifies male and female horse (*stallion, mare*)

Synexpression	Examples
Syllexification	English *bequest* syllexifies ‘give’ and ‘as inheritance’
Syngrammification	Latin *-ibus* syngrammifies ‘dative’ and ‘plural’
Circumlexification	German circumlexifies ‘desk’ (*Schreib-tisch* ‘write-table’)

Of these terms, the best-known is *colexification* (François, 2008, 2022; List et al., 2018), which has also been adopted by psychologists (e.g., Jackson et al., 2019). It was soon generalized to *coexpression* (covering both lexical and grammatical patterns; Hartmann et al., 2014), but the other terms are more novel. However, the phenomena are not novel at all: Cogrammification has often been discussed under the heading of “grammatical polysemy” or “semantic maps” (Georgakopoulos and Polis, 2018). Research on syllexification has often used the term “lexicalization pattern” (Talmy, 1985; Levin and Rappaport Hovav, 2019), and for syngrammification, linguists have often used the term “cumulative exponence” (e.g., Igartua, 2015).

While the study of lexification patterns has become much more popular over the last two decades (using the term “lexical typology,” e.g., Koch, 2001; Koptjevskaja-Tamm et al., 2016), the similarities between lexification patterns and grammification patterns have not been explored systematically. Grammatical “polysemy patterns” have typically been studied separately from lexical semantics, and inflectional “syncretism” has typically been treated in idiosyncratic ways. For syngrammification (or “cumulative exponence”), the parallels with lexical synexpression have gone unnoticed.

Apart from introducing a systematic set of terms, the present paper suggests that explanatory approaches to coxpression and synexpression patterns should be general enough to extend to both lexical and grammatical phenomena, because there is no reason to think that different causal mechanisms are at play. The reason why they have rarely been considered together has more to do with age-old traditional divisions (“lexicon” vs. “grammar,” cf. Haspelmath, 2024) than with substantive differences.

## 2. Many-to-one correspondence

As a first approximation, both coexpression and synexpression can be described as deviations from the canonical ideal of a one-to-one correspondence between meanings and shapes (or forms; this ideal has been called *biuniqueness*, e.g., Dressler, 2005). They are defined as follows:^1^

(1) a. coexpression: one minimal shape has two different meanings *in two different situations*
b. synexpression: one minimal shape has two meanings *simultaneously*

What coexpression and synexpression share is that they show a many-to-one mapping between meanings and shapes, but the many-to-one relation concerns different contexts in coexpression and the same context in synexpression. Other examples of deviation from biuniqueness are synonymy (one-to-many mapping between meanings and shapes in different situations) and multi-exponence (simultaneous one-to-many mapping between meanings and shapes),^2^ but these will play no role here.

Both coexpression and synexpression can concern either root morphs or grammatical morphs (a *morph* is a minimal form). The mapping of meanings onto root morphs is called *lexification*, and the mapping of meanings onto grammatical morphs is called *grammification*.^3^ There are thus two subtypes of coexpression (colexification and cogrammification), and two subtypes of synexpression (syllexification and syngrammification), as seen in (2a-b) and (3a-b).

(2) subtypes of coexpression

a. colexification (coexpression of two lexical meanings)

e.g., German*Tasche*‘bag; pocket’

English*go*‘go by foot’ (German *gehen*); ‘go by vehicle’ (German *fahren*)

b. cogrammification (coexpression of two grammatical meanings)

e.g., German*ich singe*‘I am singing (progressive); I sing’ (habitual)

English*to Washington*‘in the direction of W. (allative); for W. (dative)’

That German *Tasche* (or the German Present Tense) should be associated with two different meanings is suggested by an English perspective, and that English *go* (or the English preposition *to*) should be associated with two different meanings is suggested by a German perspective. It is such a comparative perspective that is adopted throughout this paper.

(3) subtypes of synexpression

a. syllexification (synexpression of two lexical meanings)

e.g., German*Onkel* ‘mother’s brother’ (cf. Swedish *mor-bror*)

English*kitten*‘young cat’ (cf. German *Katzen-junges* [cat + young])

b. syngrammification (synexpression of two grammatical meanings)

e.g., Latin*omn-ibus*‘to all’ (*−ibus* = ‘dative + plural’)

French     *décriv-ai-ent* they were describing’ (*−ai* = ‘imperfective + past’)

It is important to stress that the meanings that we are talking about in the present context are *comparison meanings*, i.e., semantic comparative concepts designed for cross-linguistic comparison. They are not (necessarily) meanings of particular languages in terms of which these languages are described. For example, saying that English *go* coexpresses the meanings ‘go by foot’ and ‘go by vehicle,’ as in (2a), is not motivated by the need to describe English. The verb *go* simply means ‘move in a particular direction,’ regardless of the mode of transportation.

Now is this way of talking about English unduly influenced by German, which distinguishes between *gehen* and *fahren* (cf. 2a)? No, because we generally need to distinguish between language-particular meanings (perhaps described in a language-particular metalanguage) and general meanings (or comparison meanings) that can be applied to all languages in the same way. The comparison meanings ‘go by foot’ and ‘go by vehicle’ could be applied to any language, and they are not privileged in any way (I merely used them here for illustration because German is a well-known language). As I noted in Haspelmath (2010, p. 668, 2018, p. 88), comparative concepts are (logically) distinct from descriptive categories not only in phonology and morphosyntax, but also in semantics. Just as English *go* probably does not have multiple meanings,^4^ German *Tasche* is not polysemous in German: While it is translated as ‘bag’ or ‘pocket’ in different situations, for German speakers, there is probably a unitary concept of ‘Tasche’ that neutralizes the distinction that English makes.^5^

When we say that A coexpresses B, or that A synexpresses B, A can be either a language or a minimal form. Thus, François (2008, p. 170) says that “a given language is said to colexify two functionally distinct senses if, and only if, it can associate them with the same lexical form,” with a language as the subject of colexification. By contrast, Schapper et al. (2016, p. 361) say that the word *rowa* in the New Guinean language Duna “colexifies ‘tree,’ ‘firewood’ and ‘fire,’” using a noun as the subject of colexification. Similarly, we could say that English *kitten* syllexifies ‘cat’ and ‘young,’ or that the English language syllexifies these two meanings with its form *kitten*.

As there are no limits on the kinds of meanings that might serve as comparison meanings, no absolute statements about particular languages can be made. The concepts of coexpression and synexpression make sense only in a comparative context.^6^

In the next four sections, I will describe coexpression (§3) and synexpression (§5) in some more detail, and along the way provide a brief introduction to coexpression universals (§4) and synexpression universals (§6). It is only universals that we can hope to explain in a general way, so these universals are crucial for the explanatory suggestions that will be the subject of §7.

## 3. Coexpression and its subtypes

### 3.1. Definitions of key terms

The key terms are defined straightforwardly as in (4)–(6). It can be noted that these definitions are completely parallel to the definitions of synexpression and its subtypes (see §5 below).

(4) **coexpression** (of two meanings A and B):

= expression of either A or B by a minimal form or a construction

(5) **colexification** (of two meanings A and B):

= expression of either A or B in a root

(6) **cogrammification** (of two meanings A and B):

= expression of either A or B in a grammatical marker

The term *colexification* was coined by François (2008), and it was generalized to *coexpression* by Hartmann et al. (2014). The two main types of linguistic expression are expression by lexical forms (or *lexification*) and expression by grammatical forms, which we can call *grammification* (a neologism introduced here for terminological symmetry). Then, by analogy with colexification, the availability of different meanings in a grammatical marker can be called *cogrammification* (rather than “grammatical polysemy,” as in much earlier work such as Emanatian, 1991; Goddard, 2003).

For simplicity, this paper only mentions situations where two meanings are (or can be) expressed by a minimal form, but of course there can be more than two meanings, both in coexpression patterns and in synexpression patterns.

### 3.2. Some earlier terminology

As I mentioned, lexical and grammatical coexpression patterns have often been described with the term *polysemy*, which goes back to Bréal (1897) (e.g., Nerlich et al., 2003; Gries, 2015). But as we saw, there is often no reason to think that the forms that are compared across languages must have several meanings from a language-internal perspective, so *coexpression* is a better term for contexts in which different languages are compared. It is well-known among semanticists that distinguishing between polysemy (or ambiguity) and indeterminacy (or vagueness) is rather difficult (e.g., Geeraerts, 2001; Riemer, 2010: §5.3), so it is often more practical to ignore this distinction, as is done with the term *coexpression* (and especially *colexification,* when applied to word meanings).^7^ Another term that linguists sometimes use is *multifunctionality*, where the vague term *function* seems to stand for a comparison meaning. Finally, Bybee et al. (1994, p. 44) talk about different *uses* of tense-aspect-mood forms in the world’s languages, by which they also mean comparison meanings.

Another widely used term in comparative semantics is *categorization*: Linguists often say that different languages *categorize* particular domains (e.g., the human body, kinship relations, perception) differently by their lexical items (e.g., Koptjevskaja-Tamm et al., 2016, p. 434), or that they have different *semantic categories* (Evans, 2011: §2). And often they say that meanings are *lexicalized* in different ways in different languages. As these terms (categorization, lexicalization) are used with other senses elsewhere, it would be clearer to talk about different *lexifications* in different languages. (And more generally, “lexical typology” is perhaps better called *lexification typology*).

For grammatical markers, the term *syncretism* has been used for quite some time in a synchronic sense (e.g., Plank, 1991; Baerman, 2007). For example, Latin *homo* ‘human, man’ has the inflectional paradigm in (7). The singular has five different forms, but in the plural, nominative and accusative are “syncretized” (i.e., they are cogrammified), as are dative and ablative.

(7)singularplural

nominative *homo    homin-es*

accusative  *homin-em  homin-es*

genitive *homin-is homin-um*

dative *homin-i homin-ibus*

ablative *homin-e   homin-ibus*

The term *syncretism* has also been used for coexpression patterns of grammatical markers beyond inflectional paradigms (e.g., complementizers, Baunaz and Lander, 2018; voice markers, Bahrt, 2021), but it is a rather odd and opaque term (originally referring to mixtures of beliefs), and it can easily be replaced by *coexpression*, or more specifically *cogrammification*. (Instead of saying that a marker is syncretic, we can say that it is *coexpressant*, but it should always be made clear whether this is meant with respect to general meanings or language-particular meaning distinctions.)

### 3.3. Some further terminology

The opposite of coexpression can be called “disexpression,” and in particular, we can talk about *dislexification* (François, 2022):

(8) **dislexification** (of two meanings A and B):

= expression of A and B by two different roots

This term can only be used about languages; for example, we can say that German dislexifies ‘go by foot’ (*gehen*) and ‘go by vehicle’ (*fahren*). Another term is *partial colexification* (List, 2023):

(9) **partial colexification** (of A and B)

= expression of A and B in composite forms that contain the same root

An example is German *Tuch* ‘cloth,’ which partially colexifies ‘towel’ (*Hand-tuch* ‘hand-cloth’), ‘shroud’ (*Leichen-tuch* ‘corpse-cloth’) and ‘sheet’ (*Bett-tuch* ‘bed-cloth’).

## 4. Coexpression universals

Coexpression universals have been discussed both for lexical and for grammatical morphs. The best-known type of diagrammatic representation is the coexpression diagram (“semantic map”), but for inflectional patterns, underspecification is the most common representation, and some generative approaches use functional sequences.

### 4.1. Semantic maps as coexpression diagrams

While colexifcation patterns have become quite famous also among psychologists in recent years (e.g., Jackson et al., 2019; Xu et al., 2020; Brochhagen and Boleda, 2022), cogrammification was the first prominent domain of coexpression studies in linguistics. Georgakopoulos and Polis (2018) survey the tradition of “semantic map studies” going back to work such as Anderson (1982), van der Auwera and Plungian (1998), and Cysouw et al. (2010).^8^ Most of this work deals with meanings of tense-aspect-mood markers, case markers, connectives and other grammatical markers, but Haspelmath (2003) already mentions the colexification patterns as highlighted by Hjelmslev (1953). Since the term “semantic map” has rather different senses outside of linguistics, it seems best to call such representations “coexpression diagrams”:

(10) **coexpression diagram**

= a graphic representation of coexpression relationships

**Figure 1:**
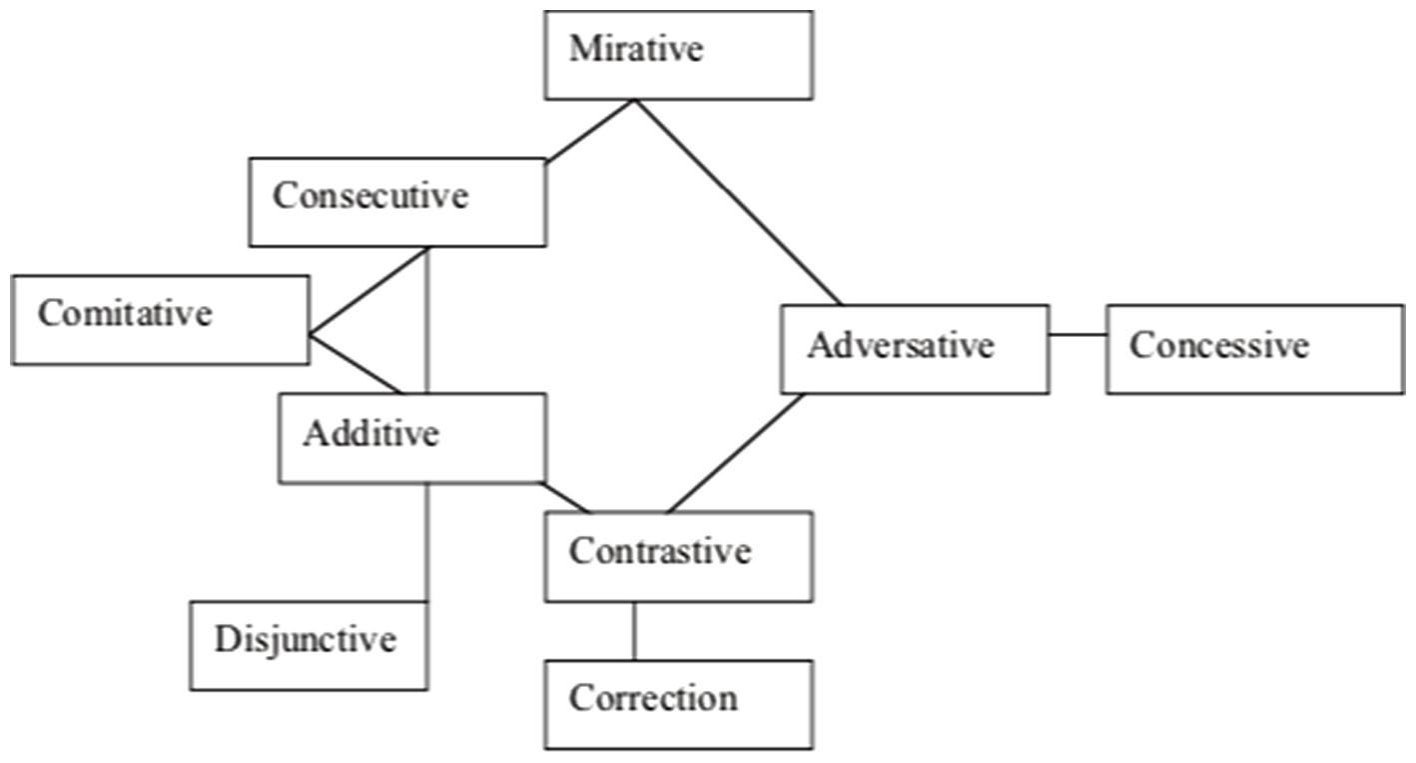
A semantic map for coordinating connectives.

Two different types of representation have become well-known: On the one hand, *connectivity diagrams*, i.e., simple graph representations with connecting lines showing possible coexpression sets [as in Figure 1, from Malchukov, 2004]. These diagrams have been used for small datasets where semantic analysis is at the center of attention.

**Figure 2:**
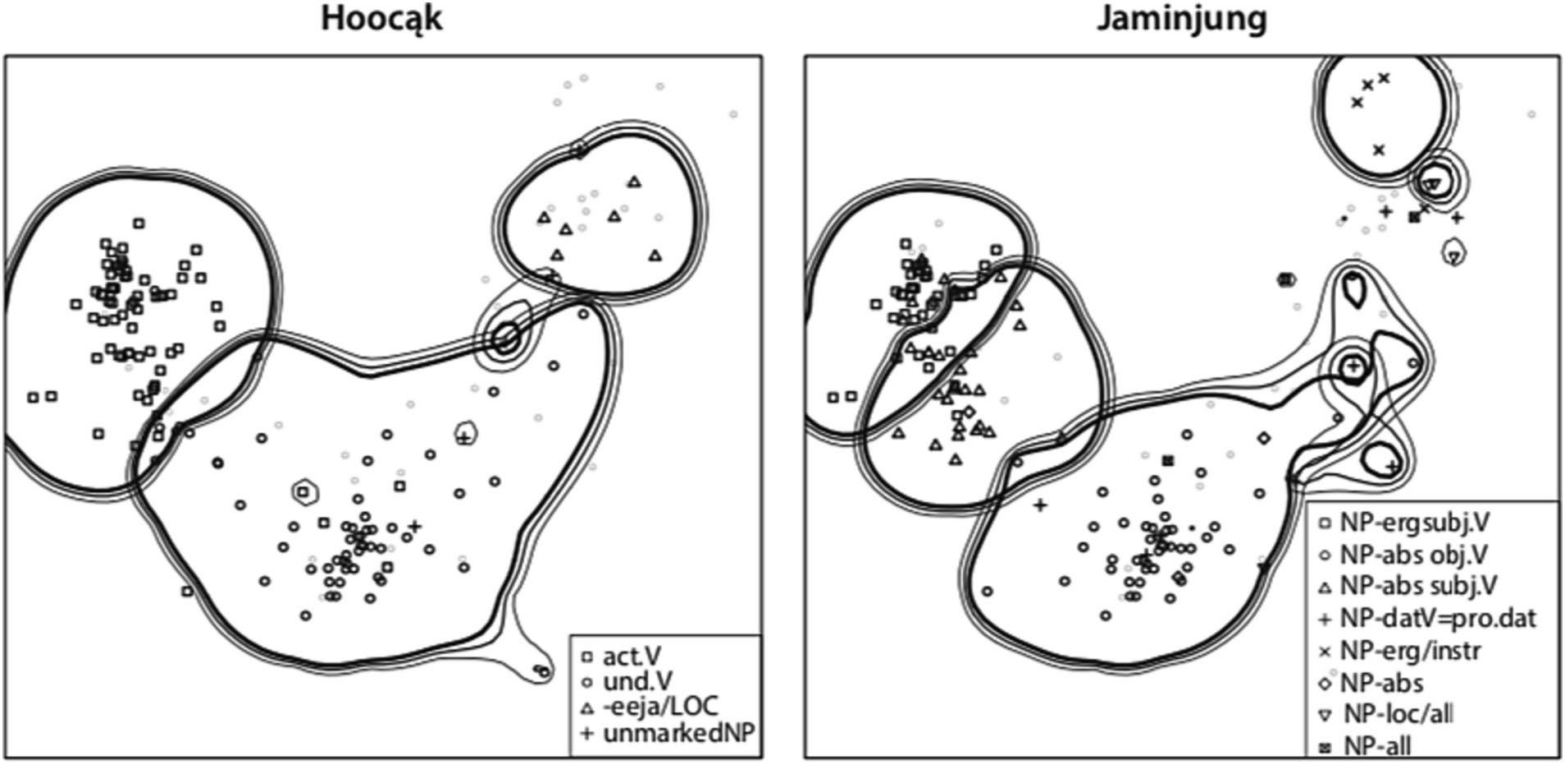
Ranges of argument-coding markers in Hoocąk and Jaminjung.

On the other hand, linguists have used *proximity diagrams*, based on clustering techniques such as multidimensional scaling. Figure 2 shows the range of uses of argument-coding markers in two different languages, based on the valencies of 80 comparable verbs in three dozen languages (Hartmann et al., 2014, p. 473). In such proximity diagrams, spatial closeness of two dots indicates that the meanings they represent are often coexpressed.

If a coexpression diagram is based on a (reasonably) representative set of languages, it embodies claims about universals of coexpression: Those meanings that are linked by a connecting line (or are in close proximity) are claimed to be very likely to be coexpressed in some language. In Haspelmath (1997) and some subsequent work (e.g., Hengeveld and van Lier, 2010), they are therefore called *implicational maps*. For example, the map in Figure 1 entails the implicational universal that if a connective marker has an adversative and a correction meaning, it also has a contrastive meaning.

### 4.2. Underspecification

When there is syncretism or coexpression within an inflectional paradigm, a common way of representing the pattern is by means of underspecification. For example, instead of positing two different suffixes *-(e)n* (1st and 3rd plural) in German verb inflection (as shown in 11), we can posit the underspecified suffix *-(e)n* [+pl] (see 12). Since the suffix *-t_2_* is more highly specified, it takes precedence over *-(e)n* in the second person plural.

(11) German verb inflection

**Table:** 

		prs	pst	
SG	1	*lach-e*	*lach-te*	‘(I) laugh, laughed’
	2	*lach-st*	*lach-te-st*	‘(you) laugh, laughed’
	3	*lach-t*	*lach-te*	‘(s/he) laughs, laughed’
PL	1	*lach-en*	*lach-te-n*	‘(we) laugh, laughed’
	2	*lach-t*	*lach-te-t*	‘(you) laugh, laughed’
	3	*lach-en*	*lach-te-n*	‘(they) laugh, laughed’

(12) underspecification analysis (following Wunderlich, 1996, p. 95)

**Table:** 

*-e*	[+1]/−pst
*-st*	[+2]
*-t_1_*	[]/−pst
*-(e)n*	[+pl]
*-t_2_*	[+2, +pl]
*-te*	[+pst]

Underspecification allows formally elegant analyses of syncretism patterns in particular languages, but the cross-linguistic significance of such analyses is sometimes unclear (see Baerman, 2007; Hein and Weisser, 2021 for recent overviews of the literature in syncretism). These analyses are relavent to universals only if strong assumptions about an innate feature inventory are made.

In a different research tradition, Greenberg (1966) included inflectional syncretism in his discussion of “markedness” asymmetries,^9^ observing that it is especially in “marked” environments (such as plural or past tense) that inflectional syncretism occurs [both are illustrated by the German paradigm in (11)].

### 4.3. Functional sequences

In yet another tradition (with its source in generative syntax), coexpression universals are expressed by the notion of a *functional sequence*, a set of hierarchically arranged functional categories that are assumed to be given in advance, presumably innately (see Cinque, 2013). For example, Baunaz & Lander (2018, p. 8) consider demonstratives, complementizers, relativizers, *wh*-pronouns and indeterminate pronouns, and posit a functional sequence “Dem > Comp > Rel > Wh > Indet” to explain the widely observed coexpression patterns in European languages, e.g., English *that* (Dem/Comp/Rel), Spanish *que* (Comp/Rel/Wh), Serbo-Croatian (Comp/Rel/Wh/Indet) *što*, German *was* (Wh/Indet). *De facto*, this approach is similar to implicational maps, but it is practiced by linguists from different communities (see also Hein and Weisser, 2021: §4).

## 5. Synexpression and its subtypes

### 5.1. Definition of key terms

The next key terms are defined straightforwardly as in (13)–(15), in a way that is fully parallel to the definitions of coexpression and its subtypes (see §3.1 above).

(13) **synexpression** (of two meanings A and B):

= expression of both A and B by a minimal form or a construction

(14) **syllexification** (of two meanings A and B):

= expression of both A and B in a root

(15) **syngrammification** (of two meanings A and B):

= expression of both A and B in a grammatical marker

The initial examples of syllexification that I gave in Table 1 and in (3) above were English *bequest* ‘give + as an inheritance,’ and *kitten* ‘cat + young.’ As the notion of syllexification is less familiar than colexification, I give more examples of syllexification in Table 2, with counterparts in other languages showing that many meanings need not be expressed as in English. Thus, the German *Fahr-rad* [ride+wheel] corresponds to syllexified *bike* in English, and the French *fauteuil* corresponds to the circumlexified *arm + chair* in English.

**Table 2:** English syllexifications, and English composite expressions corresponding to syllexifications elsewhere.

		*desk*	German	*Schreib-tisch*	[write+table]
		*nostril*	German	*Nasen-loch*	[nose+hole]
		*Leave*	German	*weg-gehen*	[away+go]
		*Uncle*	Swedish	*mor-bror*	[mother+brother]
		*eleven*	Japanese	*juu-ichi*	[ten+one]
		*boy*	Tagalog	*batang lalaki*	[child male]
French	*fauteuil*	*arm + chair*			
French	*cahier*	*note + book*			
Russian	*vanna*	*bath + tub*			
German	*Enkel*	*grand + child*			
Turkish	*fırın*	*bak-ery*			
Chinese	*mèimei*	*younger + sister*			

Syllexification can also be exemplified by suppletive comparatives such as English *worse* (‘more + bad’) and French *mieux* (‘more + good’; cf. *bon* ‘good’), or by suppletive ordinals such as Russian *pervyj* (‘one + − th’) and English *second* (‘two + − th’).

As in the case of coexpression, when we say that a form synexpresses several meanings, we do not claim that these meanings should exist in the language in question, but we are making statements on the basis of comparison meanings. There are probably many cases of synexpression of meanings that would not be easy to render in a non-synexpressed way in the same language, as when a language has a word for ‘float,’ which means ‘move on the surface of a liquid,’ and it lacks a general word ‘liquid.’ In such cases, a syllexification view is motivated only by a comparative perspective and cannot be justified language-internally.

Syngrammification (i.e., synexpression of grammatical meanings, or cumulation) is generally taken to imply that the meanings exist independently, e.g.,

(16) a. case + number:Latin *omn-ibus* ‘to all’ (cf. 3b)

b. tense + aspect:   French *décriv-ai-ent* ‘(they) described’ (cf. 3b)

c. tense + person:  Latin *vid-i, vid-isti, vid-erunt*

‘I saw, you saw, they saw’

However, case and number are never expressed separately in Latin, and imperfective aspect is never expressed separately in French, so from a language-particular perspective, one might treat these as single meanings. This is even more the case with person + number, which are very often syngrammified, not only in person indexes (bound person forms), but also in independent personal pronouns, as illustrated in (17). Only the first person plural independent person form is usefully analyzed as containing a plural suffix (*-s*), while the other forms show synexpression of person + number.

(17) Spanishindexesindependent pronouns

1 + sg*-o/−eyo*

2 + sg*-s(te)tú*

3 + sg*-Ø/−oella*

1 + pl*-mosnosotras*

### 5.2. Some earlier terminology

The way linguists have talked about synexpression is even more varied than the terminology for coexpression. Hockett (1947, p. 333) used the term *portmanteau morph* for forms like French *au* [o] ‘to + the’ or Spanish *-é* ‘1sg + preterite’ (in *canté* ‘I sang’), and this term is still common.^10^ Another well-known term for syngrammification is Matthews’s (1972) term *cumulative exponence* (contrasting with *separative* exponence, Bickel and Nichols, 2007: §1.5.1).^11^ In Distributed Morphology parlance, cumulative exponence has often been treated in terms of “feature bundling” (e.g., Matushansky, 2006).

For syllexification, there are a number of earlier terms which are familiar, but not often thought of as technical terms. In Talmy’s (1985, 2000) famous paper about “lexicalization patterns,” he talks about *conflation* of different meanings in a single form (e.g., French *entrer* ‘go into’ conflates motion and path meanings), and he also mentions *incorporation* (used by Gruber, 1965). Ullmann (1957, 1966) distinguished between motivated and unmotivated words (e.g., German *Hand-schuh* lit. ‘hand-shoe’ vs. French *gant* ‘glove’). In Seiler (1975), the distinction was called *description* vs. *labeling* (e.g., German *Lehr-er* ‘teach-er’ vs. *Arzt* ‘doctor’). Ježek (2016, p. 7–11) distinguishes between *synthetic* vs. *analytic lexicalizations* (e.g., Italian *cenare* vs. English *have dinner*). Urban (2012, 2016) talks about *analyzability* of lexical items.

### 5.3. Some further terminology

The opposite of synexpression can be called *circumexpression*, and in particular, we can talk about circumlexification:

(18) **circumlexification** (of meanings A and B)

= expression of A + B in two roots corresponding to A and to B

For example, we can say that the meaning of French *fauteuil* ‘armchair’ (seen in Table 1) is circumlexified in English by means of the two roots *arm* and *chair*. (The term *circumexpression* is of course inspired by *circumlocution*.)

## 6. Synexpression universals

For synexpression (or circumexpression) patterns, there is no well-known type of diagram, and there is no systematic literature that studies cross-linguistic variation. For the most part, the earlier literature has not even treated syllexification and syngrammification together. While inflectional syncretism has been studied in general terms, there is little general research on cumulative exponence in inflection.

Perhaps the most ambitious claim relating to synexpression is Mańczak’s (1966, p. 84) law of differentiation:

(19) **Mańczak’s Law of Differentiation**

More frequently used linguistic elements are generally more differentiated than less frequently used elements.^12^

Mańczak extends his claims to phonology, graphemics and inflectional systems, but here I only discuss “differentiation” in lexical forms. Table 3 includes some of the examples of greater differentiation in higher-frequency items that Mańczak cites. (Mańczak generally only gives the highly frequent pairs, but here I add contrasting lower-frequency pairs in order to highlight the parallels.) For example, French differentiates by having different roots for the infinitive and the 3rd singular present tense of ‘go’ (*aller* vs. *va*), and Polish differentiates by having different roots for the cardinal and ordinal of two (*dwa* vs. *drugi*). Another way of putting this, in the present context, is to say that French *va* syllexifies ‘go’ and ‘3rd singular present tense,’ that Polish *drugi* syllexifies ‘two’ and ‘ordinal,’ and so on. By contrast, French *march-* only lexifies ‘walk,’ and Polish *dziesięć/ dziesiąt-* only lexifies ‘ten.’

**Table 3:** Syllexification in higher-frequency words (Mańczak, 1966, 1970).

Highly frequent				*Less frequent*		
English	*drink*	*drank*		*consume*	*comsum-ed*	
French	*aller*	*va*	‘go (inf/3sg)’	*marcher*	*marche*	‘walk (inf/3sg)’
French	*père*	*mère*	‘father/mother’	*directeur*	*directr-ice*	‘director’
Polish	*dwa*	*drugi*	‘two/s’	*dziesięć*	*dziesiąt-y*	‘ten(th)’
Italian	*buono*	*migliore*	‘good/better’	*nuovo*	*più nuovo*	‘newe(er)’
Russian	*idët*	*šel*	‘goes/went’	*igraet*	*igra-l*	‘play(ed)’
German	*Hengst*	*Stute*	‘stallion/mare’	*Löwe*	*Löw-in*	‘lion(ess)’

Mańczak’s discussion is largely limited to the traditional “suppletion” domain, and it has of course long been observed that inflectional suppletion is found particularly with high-frequency words (see Fertig, 1998). However, Mańczak’s Law easily generalizes much further, e.g., to manner of walking verbs, which tend to be monomorphic much more often than manner of flying verbs (Slobin et al., 2014); or to terms for individual digits, which are monomorphic more often for the hand (e.g., *thumb, pinkie*) than for the foot; or to dog breed terms (e.g., *poodle*), which tend to be monomorphic more often than horse breed terms; or to interrogative pronouns, which tend to be monomorphic for ‘who’ and ‘what,’ but composite for the less frequently used ‘why’ (e.g., French *pour-quoi*).

Mańczak was not the only author to observe these patterns, and for kinship terms, Greenberg (1966, p. 72–87) stated quite a few related universals (using “markedness” terminology). For example, ‘female parent’ and ‘male parent’ are universally syllexified (e.g., *mother, father*), but many languages circumexpress ‘sister’ and ‘brother’ (e.g., Soanish *herman-a* ‘sister,’ *herman-o* ‘brother’). For body-part terms, some pertinent observations are made by Enfield et al. (2006). However, Mańczak’s Law of Differentiation is the most general formulation of the relevant patterns, and while it has not been systematically tested, the generalization seems to be largely true for lexification patterns and worthy of further investigation.^13^ They also seem to extend to cumulative exponence in inflectional patterns, as discussed below in §7.2.

## 7. What explains the limits on coexpression and synexpression?

Finally, let us now consider a few ideas about explanations of the cross-linguistically general patterns that we have seen. Especially for synexpression patterns, there are not many works that have addressed general explanatory factors, so from this perspective, we seem to be in an early phase of research. Some authors have proposed that there is a small set of innate concepts (“semantic primes”) which are expressed by (minimal) lexemes in all languages (e.g., Goddard and Wierzbicka, 1994), and this would explain some universals of non-synexpression. For example, ‘good’ and ‘old’ are lexified in all languages, and there is no language which, say, lacks a word for ‘good’ and only has a word for ‘good and big,’ or ‘good and small,’ or ‘good and red,’ and so on. But clearly, the vast majority of words in all languages do not express primitive concepts (Goddard, 2001), so synexpression is rampant. In the following, I will discuss a few explanations of coexpression tendencies (§7.1) and synexpression tendencies (§7.2) that do not make reference to innate concepts.

### 7.1. Explaining coexpression tendencies

This subsection briefly introduces three main types of explanations for general coexpression patterns which appeal (i) to conceptual similarity, (ii) to the likelihood of semantic extension in diachronic change, or (iii) to the need for maintaining informativeness. These explanations need not be mutually incompatible.

#### 7.1.1. Conceptual closeness (or similarity) may explain coexpression

That polysemy generally affects similar meanings has always been well-recognized, of course. Haiman’s work about cogrammification went further in that it includes the general Isomorphism Hypothesis:

“Different forms will always entail a difference in communicative function. Conversely, recurrent identity of form between different grammatical categories will always reflect some perceived similarity in communicative function” (Haiman, 1985, p. 19).

Sometimes, identity of form does not seem to be associated very clearly with similarity of meaning, and Haiman (1974, p. 341) noted that if formal identity recurs across languages, this must be due to some similarity of function that linguists may have overlooked. Cross-linguistically recurring coexpression patterns may thus give us clues about semantic relatedness. Conversely, one may add in the present context, semantic similarity can explain coexpression patterns. Semantic or conceptual closeness may be interpreted quite literally as referring to mental closeness in humans in general. Thus, Croft (2001, 2003) refers to coexpression diagrams as “conceptual spaces” and suggests that they give us access to… “the geography of the human mind, which can be read in the facts of the world’s languages in a way that the most advanced brain scanning techniques cannot even offer us” (Croft, 2001, p. 364).

From a psychological perspective, Xu et al. (2020, p. 8) suggest that colexification patterns “reflect a tendency to reduce cognitive effort of association” and that “it may be relatively easy for children to learn new word meanings when they can use a highly associated, already-learned word meaning to guide their interpretation.” Gil (1992, p. 305) gives the concrete example of color terms, which coexpress similar colors, but never ‘green’ and ‘orange’: “No language would seem to have a word for the 500–610 mμ spectrum range, corresponding roughly to the union of green, yellow and orange, but excluding violet and blue (on the lower end) and red (on the upper end of the spectrum).” Gil notes that apparently we do not perceive this range as constituting a unitary color, i.e., these colors are not similar (or conceptually close) enough.

#### 7.1.2. Likelihood of semantic extension may explain coexpression

On the other hand, one may seek the explanations of coexpression patterns in the likelihood of diachronic meaning extensions. For example, the meanings ‘language’ and ‘tongue’ are very different synchronically, but words for the body part ‘tongue’ frequently extend to the meaning ‘language’ by metonymic shift. That coexpressed meanings are often related via metonymy and thus need not exhibit any particular similarity has been emphasized by Cristofaro (2010) [see also Croft’s (2010) reply in the same journal issue]. The explanation of coexpression patterns via diachronic change has also been discussed by Xu et al. (2016), under the name of “historical chaining,” for names of containers in English (*bottle*, *jar*, *box*, etc.).

This type of explanation of coexpression patterns thus appeals to diachrony and is a type of mutational explanation (Haspelmath, 2019). It is perhaps not easy to distinguish from the explanation in terms of conceptual closeness because it is of course more likely that a form will develop an additional meaning when this meaning is similar to the existing meaning(s). But one may argue that the explanation in terms of meaning extension is more general and specifies a clearer causal link than a general preference for coexpressing similar meanings (or, psychologically speaking, associated meanings).

#### 7.1.3. Coexpression is constrained by informativeness

A very plausible additional factor, apart from similarity or diachronic semantic extendability, is the need to restrict the range of meanings expressed by a form in order to maintain informativeness, or clarity of comprehension. For example, while a diachronic chain of semantic extensions such as ‘seize’ > ‘take’ > ‘have’ > ‘be obliged’ is plausible, languages will probably not tolerate expressions that can have all these meanings at the same time. A specific claim of this sort was made by König and Siemund (1999), who discussed the coexpression tendencies of reflexive markers. They found good evidence for an implicational coexpression sequence “self-intensifier – reflexive – anticausative,” such that reflexive markers may coexpress the self-intensifier meaning or the anticausative meaning. However, they may not coexpress both of them at the same time, presumably because these meanings are too different.^14^

### 7.2. Explaining synexpression tendencies

This subsection briefly mentions two types of explanatory approaches that are even more tentative than the explanations suggested in §7.1. It should be noted that from the perspective of language acquisition (and the evolutionary emergence of complex language), synexpression is actually the default that we start out with, and what needs explaining is circumexpression (i.e., non-holistic, composite expressions). Thus, especially the second type of explanation (in terms of frequency) is really an explanation of circumexpression.

#### 7.2.1. Circumexpression is characteristic of certain language types

It has been observed occasionally that languages may differ in the extent to which they use synexpression or circumexpression of lexical meanings, so this would be a typological parameter. This is not really an explanation of universal tendencies, but if true, it would be very relevant to explaining the cross-linguistic patterns. Ullmann (1953, p. 229–230, 1966, p. 222–224) suggests that German has more circumexpression than English or French, and he gives examples such as those in Table 4. Ullmann’s ideas were clearly influenced by de Saussure (1916, p. 133–134, 166), who distinguished between “lexicological” languages (such as Chinese) and “grammatical” languages (such as Sanskrit) (see Aranovich and Wong, 2023 for some recent discussion).

**Table 4:** German vs. English and French (based on Ullmann, 1953, 1966).

German		English	French
*Schlitt-schuh*	[glide-shoe]	*skate*	*patin*
*Schnitt-lauch*	[cut-leak]	*chive*	*cive*
*Hand-schuh*	[hand-shoe]	*glove*	*gant*
*Scheid-ung*	[separat-ion]	*divorce*	*divorce*
*Finger-hut*	[finger-hat]	*thimble*	*dé*
*hinein-gehen*	[inside-go]	*enter*	*entrer*

Another work that tried to typologize languages along these lines was Seiler (1975), who distinguished between “descriptive” (=circumlexifying) and “labeling” (=syllexifying) techniques for lexical expression, and who noted that Cahuilla (a Uto-Aztecan language of California) makes a lot of use of the descriptive technique (e.g., ‘stone’ is expressed as ‘that which has become hard,’ or ‘basket’ as ‘that which is woven’). A notorious case of a language that makes extensive use of “description” in rendering verbal events is Kalam, a language of New Guinea (Pawley, 1993), where, for example, ‘fetch firewood’ is translated as *am mon p-wk d ap ay-* [go wood hit-break get come put].

Subsequent work has not robustly confirmed the idea that languages as wholes differ along these lines. All languages have a lot of lexical morphs (many hundreds) and also have ways of expressing less commonly needed concepts by composite forms. These composite forms may take various forms, e.g., morphologically derived forms (German *Scheid-ung* [separat-ion]), compound forms (German *Handschuh* ‘glove’), or fixed phrasal combinations (e.g., French *doigt de pied* [finger of foot] ‘toe’; English *go up*, corresponding to French *monter*). When a concept is not expressed by a compound but by a fixed phrasal expression, it is not any less composite and circumlexifying. Ullmann seems to treat morphological compounds and fixed phrasal expressions very differently, without providing any justification for this.

However, a significant (if weak) correlation between the degree of circumexpression (or “analyzability”) and the number of phonological segments has recently been found by Urban (2016). In a study of counterparts of 160 nominal meanings in 78 languages, he finds that languages with smaller segment inventories have a greater tendency to show analyzable counterparts of his nominal comparison meanings.

For syngrammification (“cumulative exponence”), it has been claimed that this is typical for “fusional/flective languages” in general, while “agglutinative languages” do not show it (cf. Plungian, 2001: §2.3), but the various properties that are said to be characteristic of “agglutination” have not been shown to correlate with each other (Haspelmath, 2009).

#### 7.2.2. Circumexpression arises in low-frequency expressions because of a constraint on root length

It has long been well known that the length of words correlates strongly and universally with their infrequency (Zipf, 1935; Bentz and Ferrer-i-Cancho, 2016), and the same applies to elements larger than words (phrases) and to elements smaller than words (grammatical markers; Haspelmath, 2021). There is little doubt that this strong regularity is due to a pressure for efficient language systems, trading off speaker (producer) needs against hearer (comprehender) needs in an optimal way.

Languages allow us to express a very large number of different meanings, and if even the very rarely used meanings were expressed in an atomic way (i.e., not circumexpressed), we would need very long atomic expressions, i.e., very long roots. However, in all (or almost all) languages, the great majority of roots are monosyllabic or bisyllabic, and trisyllabic roots are rare. Quadrisyllabic roots (such as *asparagus*, *cassowary* or *parsimony*) are extremely rare, apparently in all languages. While bisyllabic roots may not be generally less preferred than monosyllabic roots, its is clear that longer roots are rarer the longer they are. While I do not know of a good explanation of this regularity, we can formulate it as in (20).

(20) The Root Length Constraint

Roots are preferably monosyllabic or bisyllabic, and longer roots are less preferred the longer they are.

It seems plausible that this constraint is related to our memory limitations, and there is probably psychological research addressing it that I am not aware of.^15^ However, I have not seen it discussed in linguistics, so whatever its explanation, it deserves to have a name (the “Root Length Constraint”) and it should become better known.

Given this constraint, it is no longer surprising that longer expressions tend to be composite once they exceed the two-syllable window. Consider the examples from English in Table 5, where each line gives expressions with rather similar meanings that differ in frequency of use. For cardinal numerals, it is well-known that higher numerals tend to be less frequent, so it is expected that they are longer, and after a threshold, they tend to be composite (in English, the first clearly composite numeral is *thir-teen*, while in French, it is *dix-sept* [10–6] ‘seventeen’). Very similar kinds of patterns are exhibited by the other examples in Table 5.

**Table 5:** Frequent (and short) vs. medium (and longer) vs. rare (and composite).

*two*	*eleven*	*six-teen*
*blue*	*violet*	*ultra-marine*
*at*	*under*	*be-low*
*go*	*exit/leave*	*go down*
*dad*	*uncle*	*grand-father*
*mouse*	*squirrel*	*guinea pig*
*dog*	*poodle*	*gray-hound*

Thus, once we understand the Root Length Constraint, we begin to understand the basic regularity of synexpression: When meanings are expressed very frequently, they tend to be expressed by a single root (or other morph), but when they are expressed more rarely, they tend to be expressed by multiple morphs, i.e., they are circumexpressed. This regularity is very similar to Mańczak’s Law of Differentiation in (17) above, and while Mańczak did not relate it to the Root Length Constraint, he was the first to see the importance of frequency of use for explaining the regularities of synexpression and circumexpression.

The frequency-based explanation seems to hold for syngrammification (cumulative exponence) as well. This is not so easy to illustrate with examples from well-known languages, but the forms in Table 6 give an impression on the kinds of patterns that we seem to find with grammatical markers in general. Negation tends to merge with highly-frequent modals in English (*won’t* vs. *may not*); German case suffixes originally syngrammified number and case (e.g., *-e* ‘dative singular’), but not with innovative plural markers (e.g., *-er-n* ‘dative plural,’ as in *Kind-er-n* ‘to children’); and French sometimes syngrammifies prepositions and articles, but only with high-frequency prepositions (e.g., *de* ‘from’) and the (high-frequency) definite article.

**Table 6:** Frequent (and cumulatively expressed) and less frequent (separative) markers.

	*Frequent*		*Less frequent*		
English	*won’t*		*may not*		
German	*-e*	‘to (sg)’	*-er-n*	[pl-dat]	‘to (pl)’
French	*du*	‘from the’	*par le*	[by the]	‘by the’

The frequency-based explanation of synexpression tendencies seems to extend to cases where people in different parts of the world have different “elaborations” of particular semantic domains, for ecological or cultural reasons (or both). For example, in circum-equatorial regions, people talk less about frozen water than in circumpolar regions, and as a result they tend to have fewer roots that syllexify ‘soft frozen water’ (e.g., *snow*) and ‘hard frozen water’ (e.g., *ice*), as discussed by Regier et al. (2016). In many cultures, kinship terms are used more frequently than in modern English (because kinship is more important in the culture), so it is not surprising that these languages have more distinct roots for kinship distinctions. For example, Japanese has *ane* ‘older sister’ and *imooto* ‘younger sister,’ and earlier German used to have *Vetter* ‘male cousin’ and *Base* ‘female cousin.’ In those cultures where cattle are kept as domestic animals, it is not surprising to find more syllexifications (e.g., *cow* ‘female bovine,’ *bull* ‘male bovine,’ *calf* ‘young bovine’), and in cultures where camels are kept as domestic animals, they tend to be expressed by more distinct roots syllexifying additional aspects (e.g., Arabic *ʔibil* ‘camel,’ *gˇamal* ‘male camel,’ *naqaat* ‘female camel’). Such culture-specific “elaborations” of the vocabulary have long been discussed and are not surprising, but for a full explanation, we need the Root Length Constraint and the insight that frequently used meanings are expected to be expressed by shorter forms.^16^

## 8. Conclusion

In this conceptual analysis paper, I discussed two main ways in which languages show many-to-one correspondences between meanings and minimal forms: By allowing one form to have several meanings in different situations (*coexpression*) or by combining several meanings that are expressed by one form at the same time (*synexpression*). What counts as a meaning in this context depends on the analyst’s perspective, as the meanings are chosen for the purposes of cross-linguistic comparison.

Unlike research on polysemy and detailed semantic analysis, the present perspective is thus limited to a comparative perspective. The questions are therefore: What are the general limits on coexpression and synexpression? Which universal tendencies can we identify amid all the cross-linguistic variation? I pointed to the tradition of summarizing such regularities in coexpression diagrams (“semantic maps”) (§4.1) and by underspecification of inflectional markers (§4.2), as well as to Mańczak’s Law of Differentiation (§6), which says that more frequently expressed meanings tend to be expressed by different roots.

Finally, I suggested some tentative explanations of the observed patterns. Coexpression patterns can be explained by conceptual closeness, by diachronic semantic extendability or by informativeness (§7.1), while synexpression is probably explained by frequency of use, as originally observed by Mańczak: If two meanings occur together frequently, they are more likely to be expressed jointly by a minimal form.

## Author contributions

The author confirms being the sole contributor of this work and has approved it for publication.

## Footnotes

^1^Linguistic expressions or forms are treated as pairs of a meaning and a shape here. It is more common to say that linguistic expressions or forms are form-meaning pairs, but the term *form* is then used in two different meanings (‘shape’ and ‘shape-meaning pair’). This is why I prefer *shape* to *form* in the present context.

^2^An example of multiple exponence in inflection is Modern Greek *é-kan-a-n* [pst-do-pst-3pl] ‘they did,’ where past tense is signaled by the prefix *é-* and also by the suffix *-a*. Multiple exponence is discussed in detail by Harris (2017).

^3^Meanings are associated not only with morphs (i.e., roots and markers), but often also with constructions. This is reflected in the definitions in (4) and (13) below, but otherwise I leave constructions (as well as idioms) out of consideration here for the sake of simplicity.

^4^Note that I said above that English *go* “is associated with” two different meanings. We would not say that it *has* two different meanings, because this would seem to imply two language-particular meanings.

^5^Some authors prefer *concept* to *meaning* for comparison meanings, but these two terms are typically used interchangeably. Here I use *meaning* because comparative linguists also work with phonological and morphosyntactic comparative concepts, which are not meanings. François (2008, §3.1) chooses the term *sense* for language-independent comparison meanings.

^6^Dressler (2005) says that deviations from binuiqueness are generally dispreferred in morphology, but this can only mean that syncretism and cumulative exponence is dispreferred in well-defined inflectional systems, with respect to language-particular meanings. (Since “inflection” cannot be clearly delimited in a general way, this claim is not readily testable and remains speculative.)

^7^In fact, colexification studies do not even distinguish between polysemy and homonymy (List et al., 2018, p. 279), although all semantics textbooks highlight this distinction (e.g., Riemer, 2010: 162; Pustejovsky and Batiukova, 2019: §6.5; Kroeger, 2022, who mentions the English homonyms *wave* and *waive*). But homonymy is quite uncommon, whereas differences in lexification patterns across languages are very common, so in bigger quantitative studies, homonymy plays a negligible role.

^8^Croft (2003, p. 134) called semantic maps *conceptual spaces*, which seems to imply that the meanings that are expressed differently in different languages are really universal *concepts*. However, coexpression diagrams are appropriate also when meanings are contextual contrasts of different types, as in Haspelmath’s (1997) implicational map for indefinite pronoun uses.

^9^Although this was taken up by Croft (2003, p. 95–99), there has not been much further discussion of these patterns in the subsequent literature (but see Storme, 2022).

^10^However, in general English, a “portmanteau word” is what linguists tend to call a *lexical blend*, i.e., a two-component form such as *motel* (from *motor* + *hotel*) or *brunch* (from *breakfast* + *lunch*). This makes it rather confusing that linguists use the term in a different meaning.

^11^Bickel and Nichols (2005) used the term *coexponence* as a synonym of cumulative exponence.

^12^“Les éléments linguistiques plus souvent employés sont, en général, plus différénciés que les éléments linguistiques plus rarement usités.”

^13^Mańczak (1970) provides a critical discussion of the generalizations in Greenberg (1966), making it clear that he arrived at his observations from 1966 independently of Greenberg.

^14^In addition, one might suspect that meanings cannot be coexpressed when they are very similar and contrast merely in one crucial respect, e.g., in being antonyms. Brochhagen & Boleda (2022, p. 6) approach the issue from a psychological perspective and conclude that “meanings are more likely to be expressed by the same word when they are neither too unrelated, nor too related, but just right.” This is very vague, and the explanation in terms of diachronic extendability seems to make the right prediction here: The reason we do not find colexifications of, say, ‘left’ and ‘right,’ or ‘six’ and ‘seven,’ seems to be the same as the reason why we never observe meaning shifts between ‘left’ and ‘right,’ or between ‘six’ and ‘seven.’

^15^It is not obvious why it should hold, at least in this strict way, because we do remember many complex expressions consisting of several morphs (e.g., *milk-y way, ergat-iv-ity, proport-ion-al re-present-at-ion*). But even though these are not (fully) compositional in their meanings, the fact that their components are partially recognizable may help in memorizing them. With longer roots such as *caterpillar* and *asparagus*, speakers seem to be tempted to break them up into constitutents and treat them as compounds (“cat pillow”?, “sparrow grass”?).

^16^Finally, the Root Length Constraint may help us understand why languages with smaller segment inventories tend to show more circumexpression (or analyzability; see Urban, 2016): These languages need more segments and more syllables to make the same distinctions, and because of the limited syllabic size of roots, they must make more use of composite expressions.
